# Forecasting Quoted Depth With the Limit Order Book

**DOI:** 10.3389/frai.2021.667780

**Published:** 2021-05-11

**Authors:** Daniel Libman, Simi Haber, Mary Schaps

**Affiliations:** Department of Mathematics, Bar-Ilan University, Ramat Gan, Israel

**Keywords:** limit order book, quoted depth, feed forward, deep learning, deep learning—artificial neural network, deep feedforward, deep feed forward neural network, feed forward algorithm

## Abstract

Liquidity plays a vital role in the financial markets, affecting a myriad of factors including stock prices, returns, and risk. In the stock market, liquidity is usually measured through the order book, which captures the orders placed by traders to buy and sell stocks at different price points. The introduction of electronic trading systems in recent years made the deeper layers of the order book more accessible to traders and thus of greater interest to researchers. This paper examines the efficacy of leveraging the deeper layers of the order book when forecasting quoted depth—a measure of liquidity—on a per-minute basis. Using Deep Feed Forward Neural Networks, we show that the deeper layers do provide additional information compared to the upper layers alone.

## 1. Introduction

Numerous studies confirm the vital role of liquidity in the financial markets. As detailed below, liquidity affects the market stability and trading activity. This is accomplished in a myriad of mechanisms. For instance, a lack of liquidity contributes to fluctuations in stock prices and returns. In addition, liquidity is often used by professional traders to manage risk.

In the stock market, trading activity is managed through the limit order book, which represents a collection of buy and sell orders placed by traders at a variety of price points. The best bid and best ask prices represent the current going market prices at which a placed order is expected to execute immediately or within a short amount of time. Since traders place orders at different price points, the best bid and best ask prices often fluctuate throughout the trading day. For instance, an influx of sell orders at market would quickly exhaust the volume available at the best bid price, thereby exposing the next bid layer, which becomes the best bid layer. This lowers the stock price. Thus, the deeper layers of the order book are constantly exposed throughout the trading day.

Liquidity is often measured through the best bid and best ask prices and volumes, which represent the uppermost layers of the limit order book. As a result, historically, much of the research has focused solely on these upper layers, essentially overlooking the deeper layers in the order book. However, the introduction of electronic trading systems in recent years rendered the examination and study of the deeper layers significantly more practical and thus of much greater interest, especially since multiple studies suggest that the deeper layers harbor valuable information about liquidity. Furthermore, apparently no research has been done on forecasting the quoted depth—a widely accepted measure of liquidity comprised of the best bid and best ask volumes—on a per-minute basis, and only a handful of studies examined the information potential of the deeper layers in the order book in relation to this measure.

In this paper, we set out to examine the efficacy of using the deeper, traditionally hidden layers of the order book in forecasting the quoted depth on a per-minute basis. We utilize Deep Feed Forward Neural Networks for our forecasting algorithm. Deep learning has been steadily growing in popularity, especially in academic studies, which have shown successful applications of deep learning algorithms in a variety of settings (see for example, Libman et al., 2019). Despite this, papers researching applications of deep learning methods to the financial markets are often recent and few in number.

Our results indicate the deeper layers of the order book provide some useful information in predicting the quoted depth on a per-minute basis, especially when compared to the uppermost best bid and best ask layers alone.

## 2. Literature Review

Liquidity plays an important role in the financial markets. As Autore et al. (2011), when a market lacks liquidity, stock prices often fluctuate widely during trading, with returns following suit. Multiple studies suggest that market liquidity impacts trading activity, confirming the essential role of liquidity in the financial markets. For instance, Lee et al. (1993) suggests that specialists use liquidity to manage the risk associated with information asymmetry. Pronk (2006) suggests that earnings announcement affect both the bid-ask spread as well as the quoted depth (both measures of liquidity further explained below). This information is useful to any trader, but particularly to market makers that use it to schedule trades as part of an overall trading strategy.

Many ways to measure liquidity have been offered and researched over the years. Hasbrouck and Seppi (2001) was one of the earlier studies that offered a few benchmarks to measure liquidity. These included the bid-ask spread—the difference between the best bid and best ask prices—as well as the quoted depth, defined as the sum of the volume of shares available for trading at the best bid and best ask prices. While, (Hasbrouck and Seppi, 2001), as well as later studies, offered a few other measures of liquidity, the two aforementioned measures seem to be the most widely studied. Most of the other benchmarks offered (for instance, by Chordia et al., 2000) were different manipulations of the same data, as well.

Despite the accepted role of quoted depth in measuring liquidity, to our knowledge no research has been done on forecasting this measure on a per-minute basis, which is vital for traders. In contrast, plenty of research has been done on forecasting bid-ask spreads, including Groß-KlußMann and Hautsch (2013), Cattivelli and Pirino (2019), and Curato and Lillo (2015). This is even more noteworthy in light of the fact that the research on quoted depth has often yielded conflicting results—for instance, (Hasbrouck and Seppi, 2001) found that liquidity measures exhibit few co-movements while (Chordia et al., 2000) discovered the opposite.

From the aforementioned research on quoted depth, much has focused on the uppermost best bid and best ask layers, with little attention given to the deeper layers of the limit order book. This again is in contrast to extensive research on the information content of the deeper layers and its impact on prices and the bid-ask spread, which has been the topic of extensive debate in the literature. See, for example, (Mäkinen et al., 2019; Nousi et al., 2019).

Since the introduction of electronic trading systems, some financial markets embarked on the practice of exposing the deeper layers of the limit order book, which was impractical in traditional dealer markets. Studies of the impact of these changes on the markets suggest that the deeper layers contain information that impacts trading decisions. For example, Curato and Lillo (2015) suggests that specialists leverage this information in trades, while Bloomfield et al. (2005) found that traders informed about the deeper layers place more limit orders. Madhavan et al. (2005) found that traders submitted fewer orders on the Toronto Stock Exchange once the latter decided to expose the top four layers, again suggesting that traders leverage the information about deeper layers in trading decisions. The article by Cao et al. (2009) shows that the deeper layers of the limit order book contribute to price discovery.

Furthermore, little research attempted to explore the relevance of Deep Neural Networks (DNN) for the limit order book. This is despite the fact that DNN architecture has been successfully used for regression applications in other fields. Examples include He (2014), Yu and Xu (2014), and He (2017), all of whom used DNN to forecast electricity loads. Specifically, He (2014) focused on evaluating Deep Feed Forward Neural Networks for predicting electricity loads.

Deep Feed Forward Neural Networks are a category of learning algorithms that consist of an input layer, output layer, and deeper layers of the neural network, as explained by Goodfellow et al. (2016). The output layer of the neural network is powered by the deeper layers of the neural network, which are built as a chain of non-linear functions, also known as the activation functions $f\left(\vec{v},{\vec{w}}_{i}\right)$. The deepest layer is considered the first—or input—layer, and the final, most superficial layer is the output layer. The first layer is embedded in the second layer, the second layer is embedded in the third layer, and so forth.

**Figure 1:**
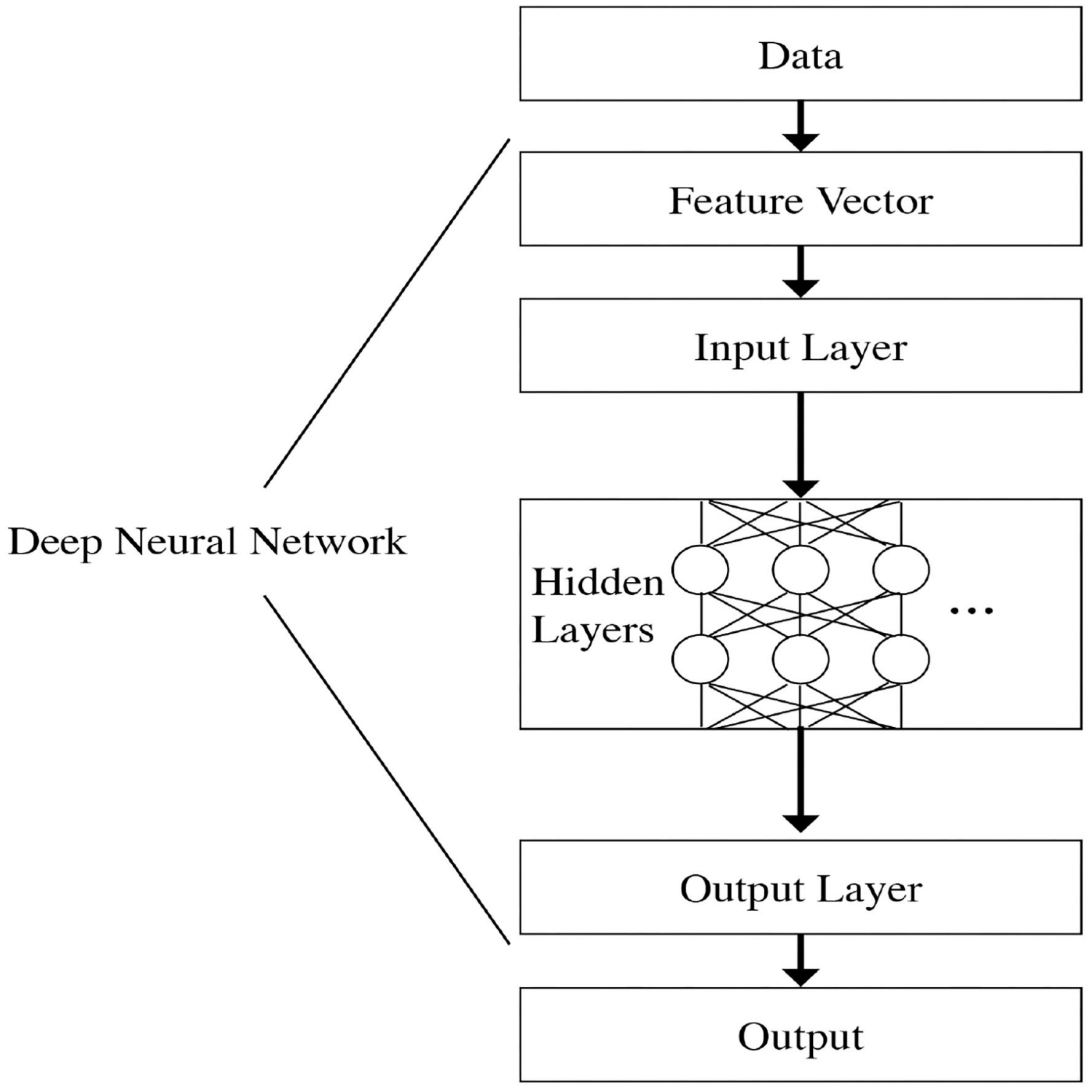
DNN structure.

Data flows from the input layer and through each layer of the network until it reaches the output layer. This represents a pattern known as “feed-forward,” since the data flows in one direction only. The input layer receives the feature vector $\vec{x}$. This structure is shown graphically in Figure 1.

Each layer is comprised of an activation function, which uses a set of parameters or weight vectors. These weight vectors are multiplied by the inputs in order to generate the output that is then fed into the next layer. This chain structure enables DNNs to execute complex input transformations when calculating the output. Adjusting the parameters or weight vectors allows the DNN to improve its prediction accuracy.

Finding the optimal transformation requires to find the best weights. This is accomplished by optimizing a cost function, also known as the loss function, using the gradient descent method. This is achieved with back-propagation (see Rumelhart et al., 1986), a dynamic algorithm that uses memory in order to reduce repetitions in the calculation of the chain rule derivatives of the large number of weights in the chain of the non-linear functions. Since the problem is not convex, there is no guarantee to find the absolute minimum, only a local one. However, results from a large body of recent deep learning research show success in a wide range of real world applications, as described by Schmidhuber (2015).

In this paper, we set out to examine the information content of the limit order book as it relates to the quoted depth. Specifically, we use a varying number of order book layers to forecast the change in log quoted depth of six stocks in the Tel Aviv Stock Exchange for the years 2012, 2013, 2017, and 2018, using DNNs for our forecasting algorithm. Our results show that the change in log quoted depth cannot be reliably predicted with the best bid and best ask layers alone, though the addition of one layer significantly improves the prediction accuracy. This suggests that at least some of the deeper layers contribute information compared with the results of using the best bid and best ask layers alone.

## 3. Methodology

We obtained the full limit order book data from the Tel Aviv Stock Exchange (TASE) for the years 2012, 2013, 2017, and 2018. This data was comprised of text files organized by date. For each trading day, there were two files: one for orders submitted and the other for transactions executed. The file detailing submitted orders also included cancellation requests.

We used these files to reconstruct the trading activity in the TASE. This enabled us to rebuild the limit order book, which was necessary for our research. Thus, we built a program that kept a running tally of all the bid and ask orders at each price level, and executed a transaction whenever there was a match, updating the market price as well as the tally at the relevant price points on both the bid and ask sides accordingly.

We accounted for each order type, as well. For instance, market orders were executed immediately at the current going price, while limit orders were executed whenever a match occurred. When an Iceberg (ICE) order was executed, the next relevant portion of the order was exposed. Special instructions, such as for stop-loss orders, were taken into account, as well. We confirmed that our program's trading activity matched the transactions executed by the TASE, using the files mentioned above. The ICE and Stop-Loss orders were introduced to TASE in 2014 (Exchange, 2014) so only the data from 2017 and 2018 included these order types.

In Table 1 are several summary statistics on the submitted orders as well as executed transactions by our six chosen stocks.

**Table 1:** Statistical summary.

**Statistic Stock ticker**	**ALHE**	**DSCT**	**ESLT**	**ICL**	**LUMI**	**POLI**
Average orders per day	5,863.39	9,788.67	7,904.84	8,610.53	6,345.25	6,804.85
Average transactions per day	401.94	1,034.44	826.66	1,746.60	1,575.16	1,492.65
Average order size	504.35	2,940.93	91.89	1,421.44	3,213.28	2,421.48
Average transaction size	482.52	2,390.21	63.12	847.50	1,908.64	1,512.45

With the limit order book thus full restored, we were able to recapture its contents at every timestamp throughout the trading day. For instance, we could see that at 9:53 a.m., the order book included certain pending orders at different price points. Thus, we could use this information to calculate the total volume of outstanding orders, both by price level (layer) as well as at all price levels combined.

For the purpose of building our model, we used the data from the reconstructed order book layers. First, we created a full representation of the order book layers, sorted by price from lowest to highest. These are shown by $\left({\vec{B}}_{i},{\vec{A}}_{i}\right)$ where ${\vec{B}}_{i}$ represents the snapshot of the bid side layers and ${\vec{A}}_{i}$ represents the snapshot of the ask side layers. After each order was submitted, our program captured snapshots of the order book on both the bid and ask side at the same time and labeled these snapshots serially. *i* represents the number of each one such serial snapshots. Thus, *i* goes from 1…*N*, where *N* indicates the number of orders for one stock for a specific date. For each day, we had *N* snapshots.

Both ${\vec{B}}_{i}$ and ${\vec{A}}_{i}$ consist of the time, price, and volume of the relevant orders submitted. Specifically, ${\vec{B}}_{i}$ can be represented as follows:

(1) $$\begin{array}{l} {\vec{B}}_{i}=\left[t_{i},\left(p_{1i}^{b},v_{1i}^{b}\right),\left(p_{2i}^{b},v_{2i}^{b}\right)\ldots \left(p_{k_{b}i}^{b},v_{k_{b}i}^{b}\right)\right] \end{array}$$

**Table:** 

where:	*t_i_*	= The time at which snapshot *i* was captured.
	$p_{1i}^{b}$	= The worst-bid price e.g., The lowest price at which a buyer agrees to buy in snapshot *i*.
	$v_{1i}^{b}$	= The volume available for trading at the worst-bid price in snapshot *i*.
	$p_{k_{b}i}^{b}$	= The best-bid price, e.g., the highest price at which a buyer agrees to buy in snapshot *i*.
	$v_{k_{b}i}^{b}$	= The volume available for trading at the best bid price in snapshot *i*.

Note that in between $p_{k_{b}i}^{b}$ and $p_{1i}^{b}$ there exist *k*_*b*_ − 2 (*k*_*b*_ excluding the best bid and worst bid layers) active price layers $\left(p_{ji}^{b},v_{ji}^{b}\right)$, e.g., prices at which volume is available for trading.

Correspondingly, ${\vec{A}}_{i}$ can be represented as follows:

(2) $$\begin{array}{l} {\vec{A}}_{i}=\left[t_{i},\left(p_{1i}^{a},v_{1i}^{a}\right),\left(p_{2i}^{a},v_{2i}^{a}\right)\ldots \left(p_{k_{a}i}^{a},v_{k_{a}i}^{a}\right)\right] \end{array}$$

**Table:** 

where:	*t_i_*	= The time at which snapshot *i* was captured.
	$p_{1i}^{a}$	= The best-ask price, e.g., the lowest price at which a buyer agrees to sell in snapshot *i*.
	$v_{1i}^{a}$	= The volume available for trading at the best ask price in snapshot *i*.
	$p_{k_{a}i}^{a}$	= The highest price at which a buyer agrees to sell in snapshot *i*, e.g., the “worst-ask” price.
	$v_{k_{a}i}^{a}$	= The volume available for trading at the “worst-ask” price in snapshot *i*.

Note that as in the case of the bid snapshots, in between $p_{1i}^{a}$ and $p_{k_{a}i}^{a}$ there exist *k*−2 active price layers $\left(p_{ji}^{a},v_{ji}^{a}\right)$ e.g., prices at which volume is available for trading.

For each order book snapshot as *i* at time *t*_*i*_, we calculated the order book snapshot 1 min ahead, represented by *i*^+^:

(3) $$\begin{array}{l} i^{+}=minj:t_{j}\geq t_{i}+1 \end{array}$$

Note that in certain cases, such as the last minute of the day, *i*^+^ did not exist and in these cases we omitted this feature.

We used this data to create the training and test samples for each of the depth configurations, e.g., number of price layers, that we wanted to use in predicting the change in log quoted depth. The number of layers was represented by *m* and varied from 1 to 9: *m* ∈ (1, 2…9).

Finally, the log quoted depth *LQD* was calculated as:

(4) $$\begin{array}{l} LQD_{i}=\log\left(v_{ki}^{b}\right)+\log\left(v_{ki}^{a}\right) \end{array}$$

When calculating the log quoted depth, we used the distance of the price of each layer from the best bid and best ask rather than the actual prices. Thus, the depth of a sample price layer ℓ from the best bid is $D_{li}^{b}=p_{ki}^{b}-p_{li}^{b}$ while the depth of a sample price layers from the best ask is $D_{li}^{a}=p_{li}^{a}-p_{1i}^{a}$.

We included all of this information in our feature vector *i* for a given number of layers used for the analysis *m*. Specifically, each sample was represented as follows:

(5) $$\begin{array}{l} \;{\vec{F}}_{i}^{m}=[log(v_{1i}^{a}),log(v_{2i}^{a})\ldots ,log(v_{mi}^{a}),\\ \;log(v_{k-m,i}^{b}),log(v_{k-m+1,i}^{b})\ldots ,log(v_{ki}^{b}),\\ D_{k-m,i}^{b},D_{k-m+1,i}^{b}\ldots ,D_{k-1,i}^{b},D_{2i}^{a},D_{3i}^{a}\ldots ,D_{mi}^{a}] \end{array}$$

**Table:** 

where:	*m*	= The number of layers the used for creating the feature vector.
	$v_{1i}^{a}$	= The ask volume available for trading at the “best-ask”.
	$v_{mi}^{a}$	= The ask volume available for trading in layer m the “worst-ask” in the feature vector.
	$v_{k,i}^{b}$	= The bid volume available for trading at the “best-bid”.
	$v_{k-m,i}^{b}$	= The bid volume available for trading in layer m the “worst-bid” in the feature vector.
	$D_{k-1,i}^{b}$	= The difference between the second “best-bid” price and the “best-bid” price.
	$D_{k-m,i}^{b}$	= The difference between the price for the m layer “worst-bid” in the feature vector to the “best-bid” price.
	$D_{2i}^{a}$	= The difference between the second “best-ask” price and the “best-ask” price.
	$D_{mi}^{a}$	= The difference between the price for the m layer “worst-ask” in the feature vector to the “best-ask” price.

This feature vector was used to predict the change in log quoted depth for a specific snapshot, or Δ*LQD*_*i*_, which was calculated as follows:

$$\begin{array}{l} \Delta LQD_{i}=LQD_{i^{+}}-LQD_{i} \end{array}$$

We ran the analysis on 10 different configurations of *m*, e.g., every number of price layers, in order to discern whether the deeper price layers of the limit order book provide any information when forecasting the change in log quoted depth 1 min ahead.

This analysis was done on a yearly basis for six stocks. For each stock and year, the data was split into three sets: train, dev, and test. We used these three sets to cross-validate the model as follows: the train dataset was used to train the model while the dev dataset was used to choose the best parameters for the neural networks. The train and dev dataset consisted of the first 10.5 (10 and a half) months of the year. To create the dev, we randomly sampled 10% of the train data. Then, we used the last 1.5 (one and a half) months of the year for the test dataset, e.g., out-of-sample. The test was used to measure the model's performance in predicting the change in *LQD*.

We trained the DNN using the Mean Absolute Error (MAE) loss function on the training test and paused the training when the MAE on the dev dataset stabilized, or stopped decreasing. Then, we calculated the MAE as well as the fraction of times in which our model correctly predicted the direction—positive or negative—of Δ*LQD* on the test dataset.

As stated above, our goal was to determine whether the deeper price layers of the limit order book provide any information when forecasting Δ*LQD*_*i*_, and if so, the ideal number of layers to use when making such a prediction. We chose to work with Feed Forward DNNs, which have shown success in other regression applications, as mentioned previously. However, because our goal was to determine whether the deeper layers contribute any additional information in the prediction, we did not focus on maximizing model performance—for example, by comparing different models or applying pre-training, as done by He (2017) and He (2014).

We used a Feed Forward Deep Neural Network (DNN) for our model with three layers in the neural network configuration. We chose this configuration as it was recommended by He (2014), who compared different DNN configurations for regression on a problem of a similar size. Our algorithm constructed nine different feature vectors corresponding to each number of order book layers used in the analysis and prediction. Thus, we had a feature vector representing zero order book layers (e.g., just the bid and ask), another feature vector representing one order book layers, another feature vector representing two order book layers, etc. Then, our algorithm fed each of these feature vectors into the DNN. This yielded nine DNNs with similar structure except for the first DNN layer, which corresponds to the length of the feature vector.

For the activation function, we chose RELU, a popular function recommended by Ryu et al. (2017), who compared the results of different activation functions for regression purposes. For all of the experiments, we used the same configurations, varying the number of layers only. This ensured that we would be able to attribute any variation in the results to the number layers of the limit order book used in the prediction.

All of our data processing code was written in Python. The DNN was implemented in Tensorflow. We ran our data processing as well as all of the machine-learning algorithms on servers provided by Google Cloud Platform.

In order to estimate the success in predicting Δ*LQD*_*i*_, we used two metrics: MAE and correct direction (CD). These formulas are shown below:

$$\begin{array}{l} MAE=\frac{1}{N}\sum_{i}|{\overset{⌢}{y}}_{i}-y_{i}|\\ \;CD=\frac{1}{N}\sum_{i}\left[\{\begin{array}{ll} 1 & sign({\hat{y}}_{i})=sign(y_{i})\\ 0 & else \end{array}\right] \end{array}$$

where:

$\hat{y_{i}}$ is the predicted value for Δ*LQD*_*i*_,

*y*_*i*_ is the actual value of Δ*LQD*_*i*_, and

*N* is the number of test samples.

## 4. Results

The full names and tickers of the stocks are detailed in Table 2.

**Table 2:** Stock ticker description.

**Ticker**	**Company name**
ALHE	ALONY HETZ
DSCT	DISCOUNT
ESLT	ELBIT SYSTEMS
ICL	ISRAEL CHEMICALS
LUMI	BANK LEUMI
POLI	BANK POALIM

Table 3 shows the average accuracy of our model when predicting the direction of the change in the log quoted depth. The average prediction accuracy is calculated as an average of the accuracy for all six stocks. The table shows the performance by the number of layers used for each year, with 0 representing the uppermost bid-ask layer. For example, our model was able to identify the direction of the change in the log size 49.33% of the time for 2012 when using only the best bid and ask. The accuracy increased to 66.86% when including one more layer and 67.11% when including two layers. The shaded cells indicate that the improvement was significant at the 95% confidence level.

**Table 3:** Accuracy.

**Layers**	**2012 (%)**	**2013 (%)**	**2017 (%)**	**2018 (%)**
0	49.33	48.31	52.24	52.52
1	66.86	65.27	66.83	67.41
2	67.11	65.46	67.19	67.91
3	67.21	65.62	67.50	68.19
4	67.25	65.60	67.57	68.22
5	67.39	65.72	67.80	68.47
6	67.59	65.75	68.11	68.55
7	67.66	65.71	68.12	68.67
8	67.82	68.92	68.32	68.79
9	67.81	68.89	68.30	68.69

*Darker shades represent lower p-values, e.g. a stronger statistical significance*.

Table 4 shows the *p*-value of the change in average prediction accuracy with each additional layer, with 0 representing the uppermost bid-ask layer. For example, for 2012, the improvement in prediction accuracy from 0 to 1 layers was significant at nearly 100% confidence, while the improvement from 1 to 2 layers was significant at nearly 99% confidence. The shaded cells highlight values lower than 5%.

**Table 4:** Accuracy *p*-value.

**Layers**	**2012 (%)**	**2013 (%)**	**2017 (%)**	**2018 (%)**
0	–	–	–	–
1	0.00	0.00	0.00	0.00
2	0.99	3.70	9.77	0.02
3	10.72	7.07	0.64	0.34
4	30.73	79.85	34.56	39.62
5	7.99	28.82	7.12	2.33
6	2.87	28.39	0.37	29.86
7	38.02	24.20	47.15	25.30
8	11.21	38.82	6.71	27.06
9	54.59	64.95	54.16	70.30

*Darker shades represent lower p-values, e.g. a stronger statistical significance*.

As these tables show, including information from the deeper layers in the algorithm does improve the prediction, compared to the upper-most layer alone (e.g., layer 0). However, the additional improvement in prediction accuracy decreases with layer depth. For instance, the inclusion of information from layer 2, when compared to layer 1, yields substantially smaller improvements than the inclusion of layer 1, when compared to layer 0. Furthermore, we see far less statistically significant values as layer depth increases.

Table 5 shows the average MAE by the number of layers used for each year, with 0 representing the uppermost bid-ask layers. Similar to Table 3, the shaded cells indicate that the improvement was significant at the 95% confidence level. Table 6 shows the *p*-value of the change in average MAE with each additional layer, with the shaded cells highlighting values lower than 5%.

**Table 5:** MAE.

**Layers**	**2012**	**2013**	**2017**	**2018**
0	0.07119	0.06398	0.06361	0.06306
1	0.05818	0.0535	0.0519	0.05125
2	0.0579	0.05308	0.05124	0.05058
3	0.05736	0.05262	0.05062	0.05029
4	0.05745	0.05255	0.05047	0.05012
5	0.05696	0.05243	0.0501	0.04981
6	0.05671	0.05225	0.04963	0.04964
7	0.05658	0.05242	0.04966	0.04958
8	0.05638	0.05125	0.04928	0.04959
9	0.05643	0.0513	0.04917	0.04955

*Darker shades represent lower p-values, e.g. a stronger statistical significance*.

**Table 6:** MAE *p*-value.

**Layers**	**2012 (%)**	**2013 (%)**	**2017 (%)**	**2018 (%)**
0	–	–	–	–
1	0.03	0.08	0.00	0.01
2	2.54	0.36	3.88	0.09
3	0.87	5.32	0.09	4.46
4	74.11	69.46	26.71	19.91
5	0.68	0.53	4.66	2.02
6	13.88	10.97	0.88	22.73
7	37.63	71.28	58.78	34.46
8	27.30	57.79	2.38	53.19
9	60.07	60.07	32.03	41.84

*Darker shades represent lower p-values, e.g. a stronger statistical significance*.

**Figure 2:**
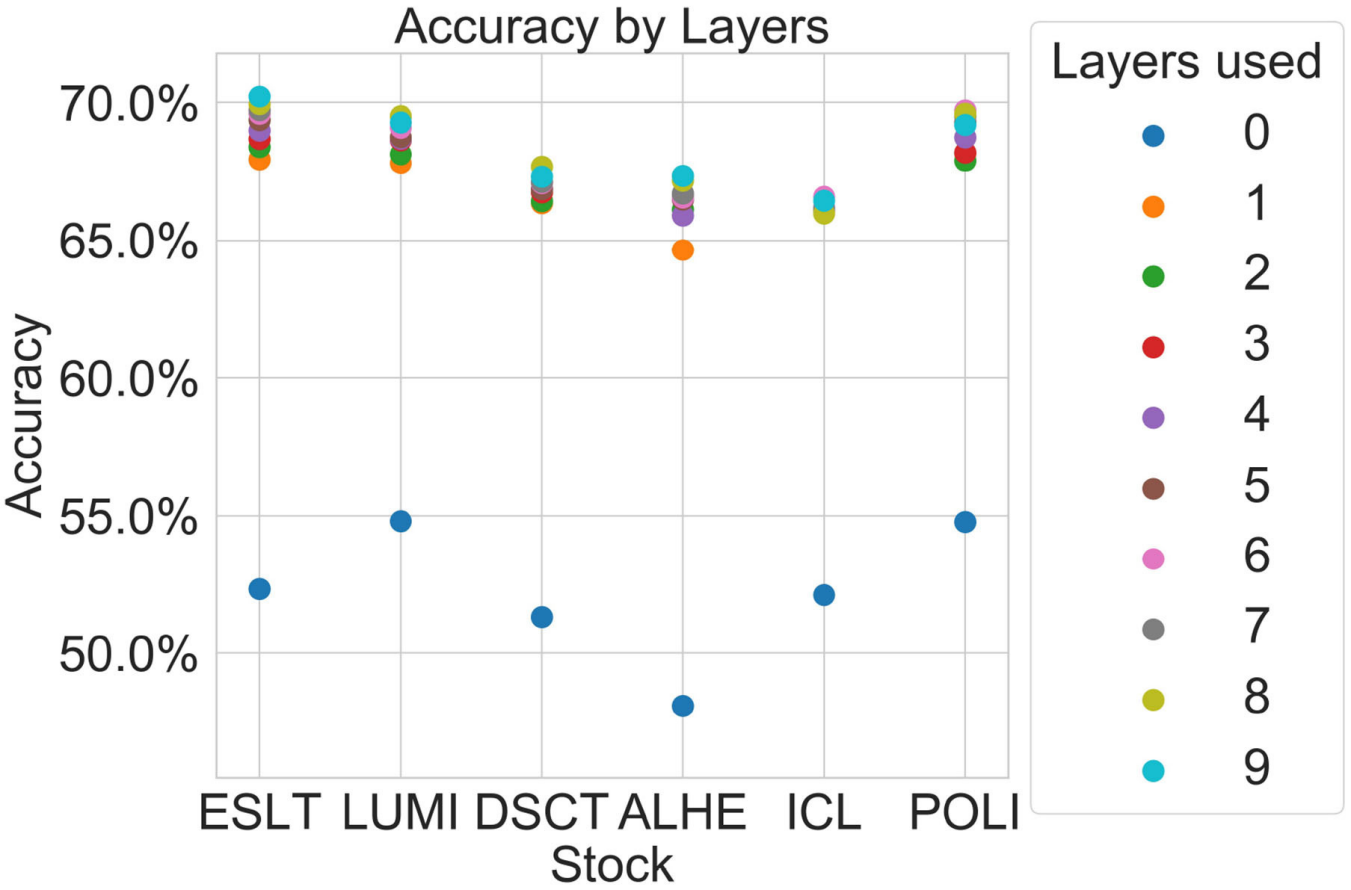
Average prediction accuracy by number of layers.

Figure 2 is a graphical depiction of the 2012 averages from Table 3, showing the accuracy by layer and stock. As illustrated in Table 3, our model cannot reliably predict the change in log quoted depth from the uppermost layer (best bid and best ask) alone. However, the addition of just one layer improves the prediction accuracy significantly. Further layers may improve the prediction accuracy further although not always. This suggests that most of the information appears in just one layer below the best bid-ask.

**Figure 3:**
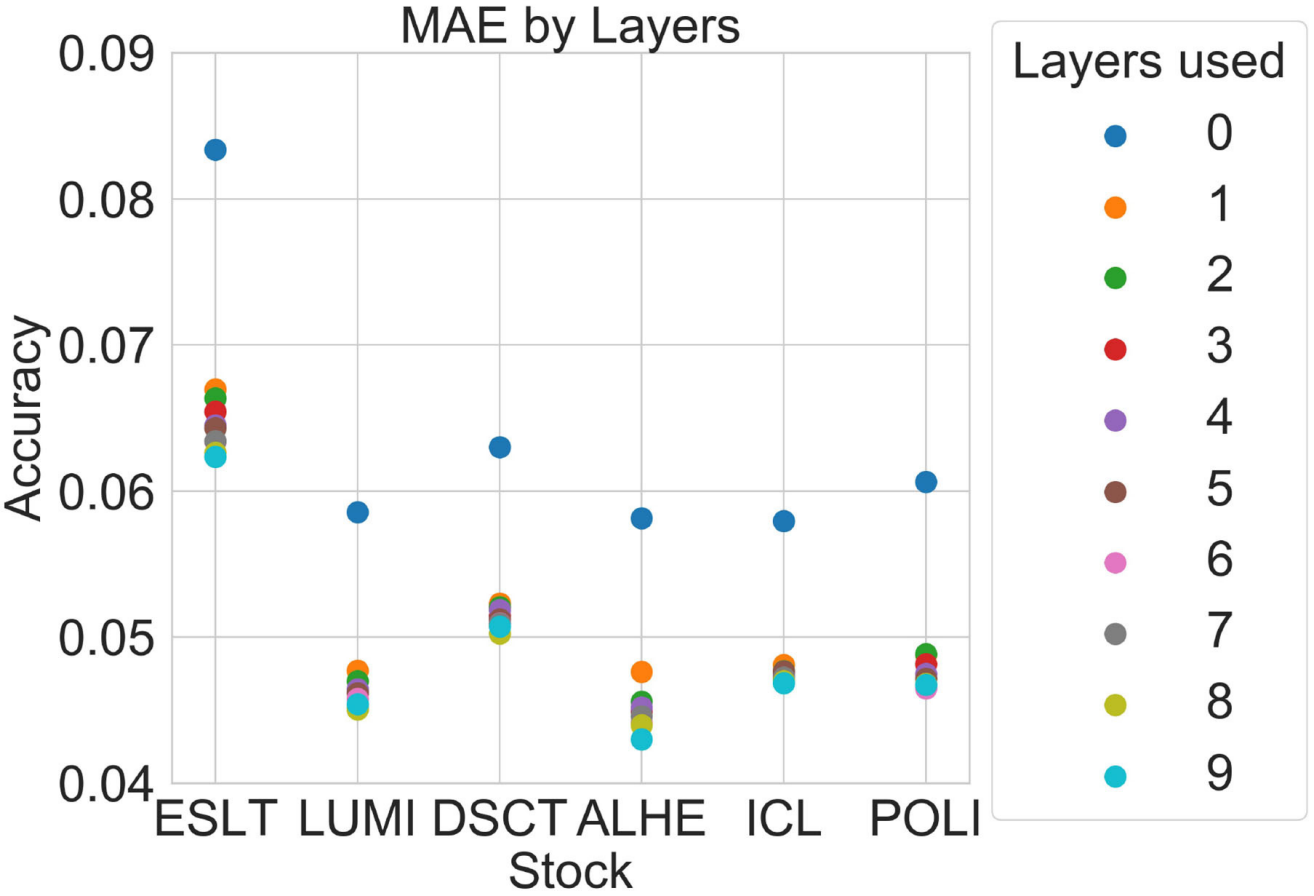
MAE by number of layers.

**Figure 4:**
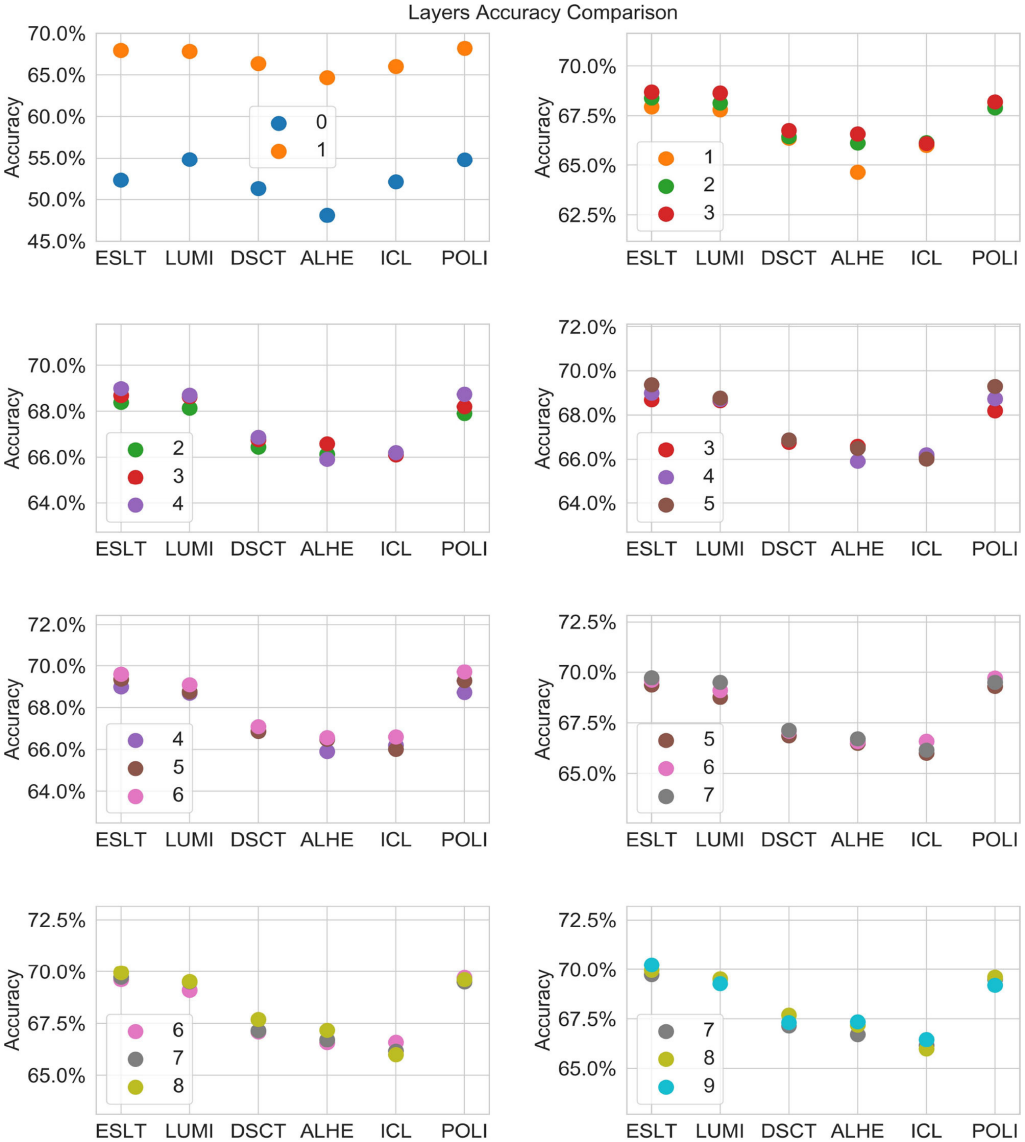
Prediction accuracy by number of layers and stock.

**Figure 5:**
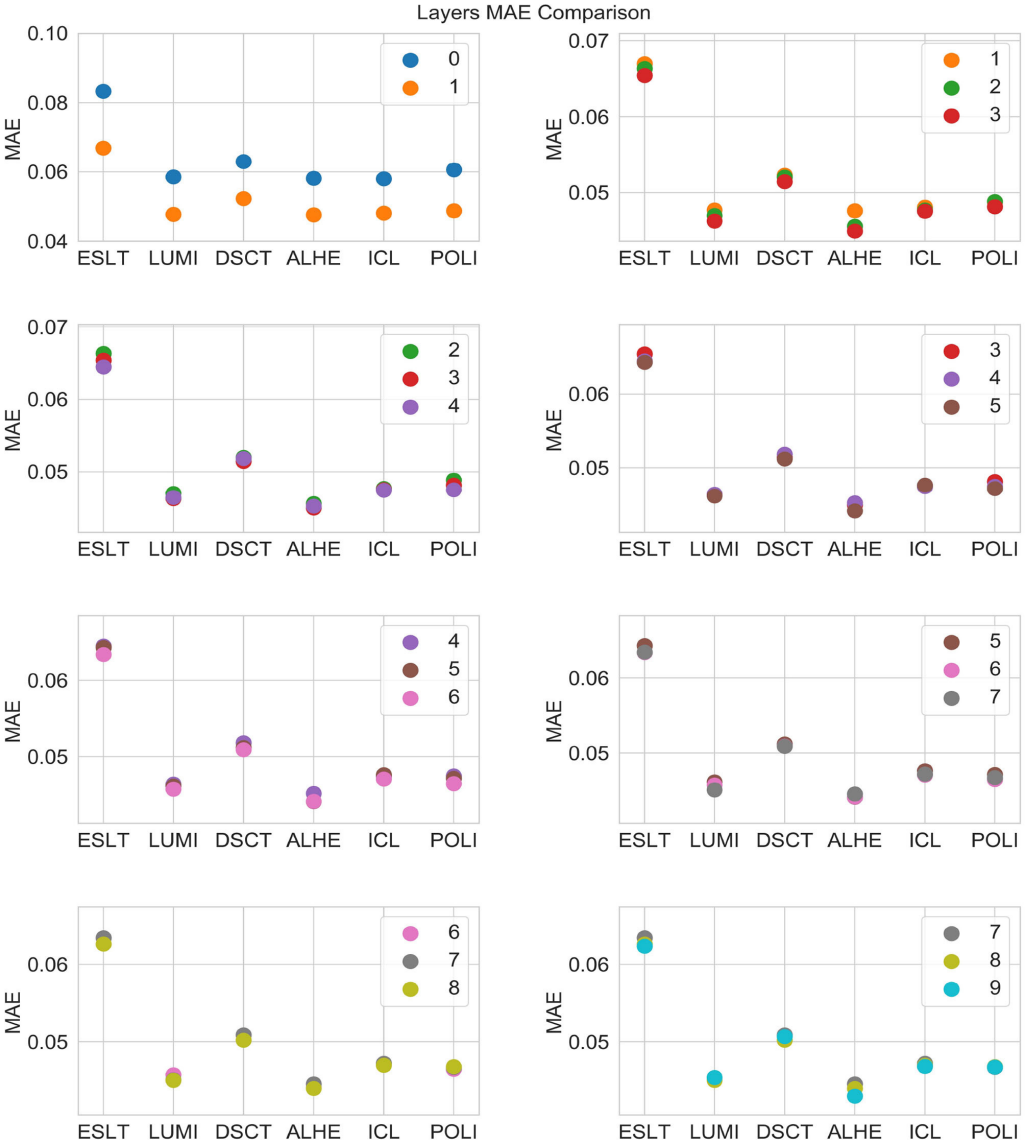
MAE by number of layers and stock.

Figure 3 is a graphical representation of the 2012 averages from Table 5. Figures 4, 5 show comparisons across subsets of layers.

## 5. Conclusion

In this paper, we set out to examine the information content of the deeper layers in the limit order book in the context of predicting the change in log quoted depth on a per-minute basis throughout the trading day. We compared the predictive power of the best bid and best ask combined with additional deeper layers with that of the best bid and best ask layers alone. Our results indicate that the change in log quoted depth cannot be reliable predicted with the best bid and best ask layers alone, and that the addition of the deeper layers substantially improves the prediction accuracy. We therefore conclude that the deeper layers of the order book possess valuable information in the context of liquidity, a finding that is supported by other studies, as well. Understanding the relevance of the deeper layers in the limit order book is especially relevant these days with the introduction of electronic trading markets, which made the open display of the deeper layers more widespread. As the practice of exposing the deeper layers of the order book becomes more prevalent, future research might consider a more detailed comparison of the predictive efficacy of different number of layers. Additionally of interest would be an analysis of multiple trading markets worldwide to examine whether factors such as trading activity or culture affect the predictive power of the deeper layers. For instance, in less busy markets such as TASE, the residual utility of each additional layer might diminish faster than in more active markets such as NYSE.

## Data Availability Statement

The data analyzed in this study is subject to the following licenses/restrictions: The dataset involves limit order book trading data from the Tel Aviv Stock Exchange (TASE). Requests to access these datasets should be directed to micky@tase.co.il.

## Author Contributions

As a primary researcher, DL was responsible for making the necessary contacts to obtain and collect the relevant data, also wrote the code to process the relevant data and research different algorithms, comparing results, and authoring most of the paper. SH offered substantial guidance throughout the entire project, proposing additional methods, and ways to experiment with the data. During the authoring stage, he initiated numerous thoughtful comments to help refine the paper and ensure its fit with the institution's academic standards. MS supervised the project, and as such she was instrumental in the ideation stage as well as providing overall direction and verifying all mathematical calculations, also facilitated access to crucial resources, including the data sources as well as several colleagues that served as valuable advisors and mentors throughout the project, and contributed essential feedback to the process that proved critical to obtaining quality results in a timely manner. All authors contributed to the article and approved the submitted version.

## Conflict of Interest

The authors declare that the research was conducted in the absence of any commercial or financial relationships that could be construed as a potential conflict of interest.
